# Complete Genome Sequence of Enterococcus faecalis Siphophage Sigurd

**DOI:** 10.1128/mra.00123-22

**Published:** 2022-03-28

**Authors:** Adam Tomaszewski, Daniel Mora, James Clark, Tram Le, Ry Young, Mei Liu

**Affiliations:** a Department of Biochemistry and Biophysics, Texas A&M University, College Station, Texas, USA; b Center for Phage Technology, Texas A&M University, College Station, Texas, USA; Portland State University

## Abstract

Enterococcus faecalis is associated with antibiotic-resistant infections, and this study presents E. faecalis siphophage Sigurd. The 41,811-bp Sigurd genome is divided into two arms defined by long convergent predicted transcription units that are separated by a bidirectional rho-independent terminator. Sigurd has a small terminase that is closely related to Bacillus subtilis *cos* phage phi105.

## ANNOUNCEMENT

Enterococcus faecalis is a common component of the human gut microbiome that is known to cause antibiotic-resistant nosocomial infections (1). The ineffectiveness of antibiotic treatments for infections caused by this pathogen necessitates the development of alternate strategies such as phage therapy. This study presents the annotated genome of phage Sigurd, which can infect E. faecalis.

Phage Sigurd was isolated in September 2019 from a wastewater influent sample collected in Port Aransas, Texas. It was propagated with soft agar overlay methods (2) using an Enterococcus faecalis isolate from mouse feces (strain Sor, provided by the University of California, San Diego [UCSD]) grown on brain heart infusion medium (Difco) at 37°C with aeration, and it showed clear plaque morphology. Phage DNA was purified using the Promega Wizard DNA cleanup system as described (3). DNA sequencing libraries were prepared as 300-bp inserts using a Swift 2S Turbo kit and were sequenced on an Illumina MiSeq system with paired-end 150-bp reads using 300-cycle v2 chemistry. The 235,068 individual raw reads were quality controlled using FastQC (www.bioinformatics.babraham.ac.uk/projects/fastqc) and trimmed with FASTX-Toolkit (http://hannonlab.cshl.edu/fastx_toolkit), yielding 164,226 reads. The reads were assembled using SPAdes v3.5.0 (4) to produce a contig with 66-fold coverage. The contig end sequences were completed by PCR (with primers 5′-TAGGCAACACTGATGGCAAAC-3′ and 5′-AGCGTTTTTCAGTCGCCAAT-3′) using phage genomic DNA as the template, with Sanger sequencing of the resulting PCR product. The Center for Phage Technology (CPT) Galaxy-Apollo phage annotation platform (https://cpt.tamu.edu/galaxy-pub) was used to annotate the genome (5–7), utilizing GLIMMER v3 (8) and MetaGeneAnnotator v1.0 (9) for gene prediction, ARAGORN v2.36 (10) and tRNAScan-SE v2.0 (11) for tRNA prediction, and TransTermHP (12) for identification of rho-independent terminators. The prediction of gene functions utilized InterProScan v5.48 (13), BLAST v2.9.0 (14), TMHMM v2.0 (15), HHpred (16), LipoP v1.0 (17), and SignalP v5.0 (18). BLAST searching was performed against the NCBI nonredundant and Swiss-Prot databases (19). Genome-wide nucleotide similarity to top BLAST hits was calculated by progressiveMauve v2.4 (20). PhageTerm was used to predict phage termini (21). All tools were run with default settings.

**FIG 1:**
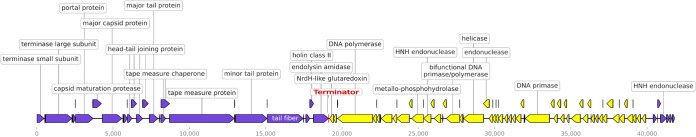
Genome map of phage Sigurd. Genes in purple are transcribed on the plus strand, and genes in yellow are transcribed on the minus strand. The predicted terminator location is indicated in red.

Phage Sigurd has a genome of 41,811 bp, with a GC content of 34.5% and a coding density of 91.5%. It has 76 annotated genes, of which 27 have predicted functions. Phage Sigurd likely uses *cos*-type packaging, based on the fact that its small terminase subunit is closely related to that of the well-studied Bacillus subtilis *cos* phage phi105 (22). The exact location of the *cos* site, however, could not be predicted by PhageTerm. The genome of Sigurd can be divided into two arms defined by long convergent predicted transcription units that are separated by a large bidirectional rho-independent terminator. Convergent transcription is presumed from the gene orientation at this location. All of the genes on either side of this point are on the same coding strand and are thus presumed to be cotranscribed, as shown in the genome map (Fig. 1). The left arm has many predicted virion structural and lysis genes, all on the plus strand. The right arm of the genome, encoded mostly on the minus strand, is dominated by novel hypothetical genes. A predicted bifunctional DNA primase/polymerase (gp35) and two HNH endonucleases were identified, one of which is near the end of the genome and may play a role in *cos* cleavage (23). As determined by progressiveMauve, Sigurd shares >70% nucleotide similarity with other *Enterococcus* phages such as vB_EfaS_Ef6.1 (GenBank accession number MK721187) and vB_EfaS_Ef6.4 (GenBank accession number MK721190). Sigurd was determined to have a siphophage morphology via transmission electron microscopy, but the image is not shown due to poor image quality.

### Data availability.

Sigurd was deposited in GenBank with accession number MZ326865. The associated BioProject, SRA, and BioSample accession numbers are PRJNA222858, SRR14095245, and SAMN18509620, respectively.
